# Cutaneous melanocytic mosaicism with associated melanoma: A case series

**DOI:** 10.1016/j.jdcr.2025.11.056

**Published:** 2025-12-29

**Authors:** Isabella Ribaudo, Elham Aldosari, Pauline Funchain, Gyorgy Paragh, Marilyn Wickenheiser, Joshua Arbesman

**Affiliations:** a Department of Dermatology, Cleveland Clinic, Cleveland, Ohio; b Stanford University, Palo Alto, California; c Roswell Park Cancer Institute, Buffalo, New York

**Keywords:** cutaneous mosaicism, dysplastic nevus, genetics, melanoma, Skin cancer

## Introduction

Cutaneous mosaicism (CMo) arises from a de novo mutation during cell migration, affecting cells in a specific area while the unaffected skin retains its normal phenotype and genotype. The study of mosaic skin patterns originated with Blaschko observations, identifying lines associated with epidermal nevi. Happle and Jackson later associated these patterns with CMo, unveiling additional patterns.^1^ The exploration of mosaicism has contributed to understanding the dynamics of various genetic diseases, paving the way for potential therapies.^2^ However, research on CMo and its correlation with melanoma risk is limited despite its potential to uncover novel drivers of melanomagenesis. Cutaneous melanocytic mosaicism (CMMo) is identified by the emergence of distinct melanocytic pigmentation patterns, visibly differing from the unaffected dominant pattern of the skin. This phenomenon, associated with melanoma development, has been reported using diverse terminology. Case reports include melanoma arising in segmental dysplastic nevi syndrome, agminated melanocytic nevi, unilateral lentigosis, and segmental speckled lentiginous nevi.^3^, ^4^, ^5^, ^6^ This case series, the largest to date, describes 6 instances of melanoma arising within different CMMo patterns. Our goal is to deepen the understanding of high-risk cutaneous patterns that may predispose individuals to melanoma. Additionally, by referencing diverse presentations of CMMo-associated with melanoma in the past, we aim to consolidate it as a singular phenomenon, enhancing its recognition in clinical practice.

## Methods

This case series involved adults diagnosed with melanoma associated with CMMo between 2008 and 2024 at Cleveland Clinic and Roswell Park Cancer Institute. Clinical data and histopathological samples were collected from each patient, and genetic testing for pathogenic variants associated with melanoma and general cancer susceptibility was conducted through Invitae Melanoma and Multi-Cancer panel testing. This study was reviewed and approved by the institutional review board of the Cleveland Clinic (IRB 17-887); informed consent was obtained from all participating patients.

## Results

**Fig 1:**
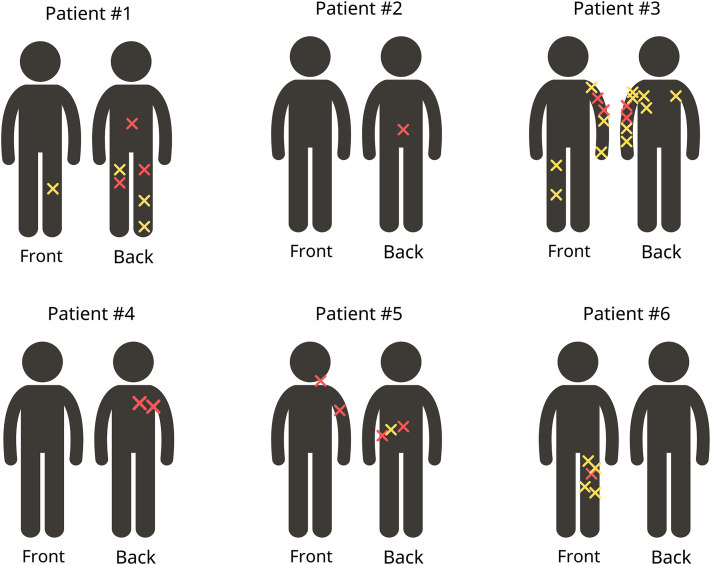
Different cases of cutaneous melanocytic mosaicism with associated melanoma. The *yellow* “X” represents a dysplastic nevus, while the *red* “X” represents a melanoma.

We identified 6 Caucasian patients with melanoma associated with CMMo (Table I). Four of the patients were male. The mean age at the time of the first melanoma diagnosis within the mosaic area was 48 years (range: 32-69). Three patients presented with a single melanoma, while 3 patients exhibited 2 or more melanomas associated with CMMo. Four patients showed a segmental pattern of CMMo, while 1 patient displayed Blaschko's narrow band mosaic pattern, and another showed a patchy pattern without midline separation (Fig 1). One patient had a family history of melanoma among first-degree relatives. Five patients underwent germline genetic testing through the Invitae Multi-Cancer and Melanoma panels without identification of any pathogenic variants (Table I). All melanomas were successfully treated.

**Table I:** Details of cases with cutaneous mosaicism with associated melanoma

Patient	Sex	Age∗	Mosaic pattern	Clinical description	Histology
1	Female	44	Patchy pattern without midline separation	Multiple asymetric brown macules on the lower extremities.	MM on the mid-back. SSM, T2a, BD: 1.75 mm on the right thigh. SSM, BD: 0.4 mm on the left thigh. ACMN on the left thigh and left medial ankle. Shave removal of multiple dysplastic nevi on left lower leg and right posterior calf. Multiple BCC.
2	Male	32	Segmental (Blaschko's lines broad bands)	Multiple oval and irregular brown macules on the right mid-lower back extending to the right lower abdomen.	SSM, T2a, BD: 1.1 mm on the mid-back.
3	Female	41	Segmental (Blaschko's lines broad bands)	Predominance of irregular brown macules present on the left upper back, extending through the posterior left arm.	Four MIS on the left upper arm. Multiple shave removal of dysplastic nevi, 9 on her left upper extremity. Multiple BCC.
4	Male	55	Segmental (Blaschko's lines broad bands)	Segmental tan patch demarcated at midline back and midline chest with underlying nodularity.	SS T2a, BD: 1.1 mm on the right medial upper back. MIS on the right lateral upper back. Multiple neurofibromas were identified in WLE.
5	Male	49	Blaschko's lines narrow bands	Three irregular brown macules on left lower back on a linear distribution.	SS, T1a, BD: 0.53 mm on the left forearm. Desmoplastic Melanoma, T2a BD: 1.1 mm on the left lateral neck. MIS, adjacent atypical junctional nevus with severe melanocytic dysplasia and MIS on the left medial back. One BCC and SCCIS.
6	Male	69	Segmental (Blaschko's lines broad bands)	Multiple round and irregular brown macules on the left buttock and left lower extremity.	SSM, BD: 0.32 mm on the lateral distal thigh. Shave removal of 4 dyplastic nevi on the left thigh. Multiple BCC and SCCIS.

∗ Age at diagnosis of first melanoma within the mosaic area. *ACMN*, Atypical compound melanocytic neoplasm; *BCC*, basal cell carcinoma; *BD*, Breslow depth (mm); *MIS*, melanoma in situ; *MM*, malignant melanoma; *SCCIS*, squamous cell carcinoma in situ; *SSM*, superficial spreading melanoma.

## Discussion

**Fig 2:**
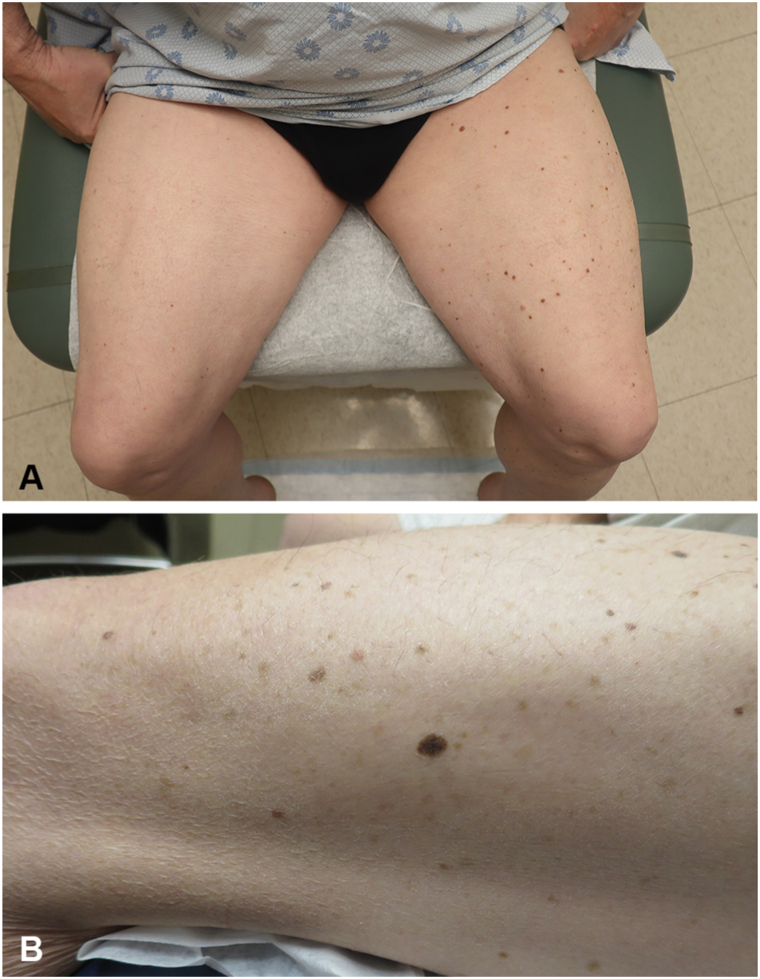
Patient #6 with a predominance of nevi on his left leg in comparison to his right leg and other areas of the body (**A**), with the patient's melanoma identified on the lateral left leg (**B**).

**Fig 3:**
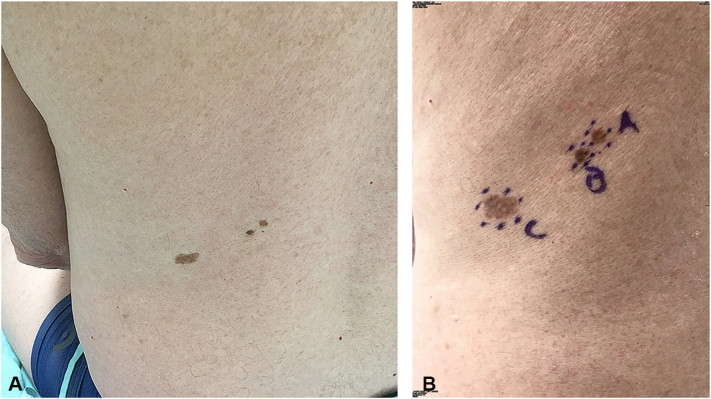
Patient #5, MIS (**A**) adjacent atypical junctional nevus with severe melanocytic dysplasia (**B**) and MIS (**C**) on left medial back is displayed.

**Fig 4:**
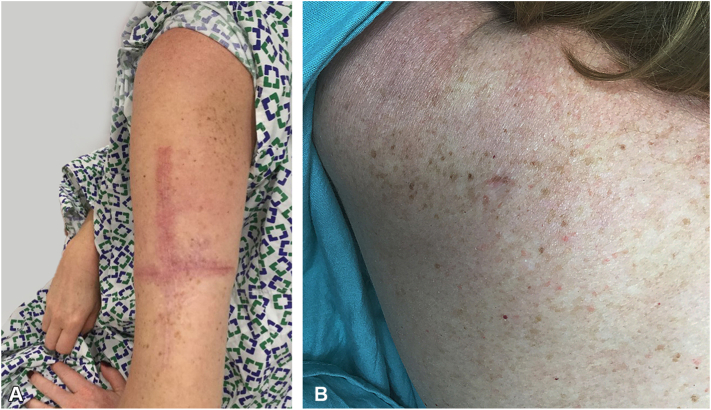
Patient #3, predominance of nevi and speckled lentiginous nevus on the left upper back (**A**), extending through the posterior left arm (**B**).

In the present case series, most melanomas arising within CMMo patterns are linked to dysplastic nevi and predominantly follow a segmental pattern; for example, patient #6 has a predominance of nevi on his left leg noted in comparison to the rest of the body, with the patient’s melanoma arising within the affected area (Fig 2). However, this is not universally the case. For instance, patient #4 presented with a demarcated segmental tan patch with underlying nodularity but no dysplastic nevi, and patient #5 exhibited 2 melanomas in situ (MIS) that coincided in their development with an adjacent atypical junctional nevus showing severe melanocytic dysplasia in a linear distribution and just millimeters apart (Fig 3). Furthermore, patient #3 CMMo manifested with not only dysplastic nevi but also a segmental speckled lentiginous nevus, reflecting how this phenomenon should not only be categorized as “segmental dysplastic nevus syndrome” or “segmental speckled lentiginous nevi” but rather should be recognized as CMMo-associated with melanoma (Fig 4). When patients with CMMo develop melanoma, it predominantly arises within the mosaic area, on average 17 years earlier than the typical age of melanoma diagnosis, even in the absence of identified pathogenic variants associated with melanoma and no family history of the disease.^7^ This pattern is consistent with findings from the literature review of previous case reports on CMMo-associated melanomas.

Melanoma associated with CMMo was initially documented in 1988, involving a 59-year-old male with multiple common and dysplastic nevi, along with 2 melanomas confined to a segmental distribution in the trunk.^8^ Since then, a small number of similar reports have appeared, describing melanoma arising within various segmental pigmentary disorders of melanocytic origin (Table II).^3^^,^^5^^,^^9^, ^10^, ^11^, ^12^ While the reported cases have apparent clinical differences, the large affected areas showing distinctly different pigmentary phenotypes with striking patterns of involvement and the emergence of melanoma in the affected area argue for their classification as CMMo. Our synopsis of the previously reported 13 cases of CMMo-associated melanomas revealed characteristic features that distinguish them from more common melanoma presentations. The average age of melanoma diagnosis in these patients is 45 years, approximately 20 years younger than the typical age of melanoma onset.^7^ On average, patients had 2 melanomas arising within the mosaic area. The most common histologic subtype was superficial spreading melanoma. Interestingly, 20 of CMMo-associated melanomas in this review arose from a nevus (70%), a rate markedly higher than the 30% typically expected.^14^ Segmental mosaicism following Blaschko’s lines (broad bands) was the most prevalent pattern, observed in 10 cases (76%), with 1 case each of lateralization and checkerboard patterns. Only 3 patients (23%) had melanomas located outside the mosaic area. Only 1 reported case included genetic testing, which yielded negative results.^11^ The consistent characteristics among patients, notably that melanomas predominantly arise within the mosaic area at a younger age than typically expected, even without a family history of the disease underscores the importance of recognizing CMMo as a singular entity for both clinicians and researchers.^7^

**Table II:** Previously reported cases of cutaneous melanocytic mosaicism associated with melanoma

Article	Age	Sex	FHM	Number of melanomas	Histology	Melanoma arising from a Nevus	Clinical description	Mosaic pattern	Melanoma(s) outside mosaic area
Quadrant distribution of dysplastic nevus syndrome^8^	59	M	No	2	#1: SSM, BD: 1.7 mm. #2: SSM, BD: 0.6 mm.	Yes	Dysplastic nevi and lentigines along with 2 melanomas on the left upper quadrant.	Segmental (Blaschko's lines broad bands)	No
Unilateral dysplastic nevi associated with malignant melanoma^9^	41	M	No	1	SSM, BD: 8 mm.	Yes	Multiple dysplastic nevi dorsally on the patient's left upper quadrant associated 1 melanoma.	Segmental (Blaschko's lines broad bands)	No
Melanoma(s) associated with a quadrant or segmental distribution of atypical melanocytic nevi^5^	30	F	No	2	MM, BD: 0.6 mm.	Yes	Numerous clustered melanocytic skin lesions on her left thigh associated 1 melanoma.	Segmental (Blaschko's lines broad bands)	Yes
Multiple melanomas arising in a partial unilateral lentiginosis^10^	25	F	No	6	#1 - 6: MIS.	Unspecified	Several hyperpigmented macules were present between the submammary skin fold and upper thigh, associated with 6 melanomas. The right side of her body showed no pigmented spots.	Left Lateralization	No
Bilateral segmental lentiginosis associated with malignant melanomas^3^	86	F	No	2	#1: SSM, BD: 0.7 mm. #2: MIS.	Unspecified	Multiple small lentigines on the left side of the face, right arm, right side of the trunk and left leg with midline stoppage. Two melanomas in affected area.	Checkerboard	No
Primary cutaneous melanoma arising in agminated melanocytic nevi^11^	32	M	No	2	MIS	Yes	Cluster of melanocytic lesions on his left chest associated 1 melanoma.	Segmental (Blaschko's lines broad bands)	Yes
A case of malignant melanoma arising from an acquired agminated melanocytic naevus^12^	36	F	No	1	MM, BD: 4.4 mm.	Yes	Cluster of naevi in the upper portion of right arm associated 1 melanoma.	Segmental (Blaschko's lines broad bands)	No
Melanoma arising in a large segmental speckled lentiginous nevus: Case series of 4 patients^4^	38	F	No	1	SSM and nevoid, BD: 2.5 mm.	No	Multiple pigmented lesions and cafe-au-lait spots confined to the right lower limb associated 1 melanoma.	Segmental (Blaschko's lines broad bands)	No
	20	F	No	1	SSM, BD: 0.70 mm.	Yes	Multiple pigmented lesions and cafe-au-lait patches on the left and right lower limb up to the umbilicus anteriorly and L1 posteriorly associated 1 melanoma.	Patchy pattern without midline separation	No
	47	F	No	2	#1: SSM, BD: 0.75 mm. #2: SSM, BD: 0.70 mm.	Yes	Multiple solar lentigines and papular pigmented lesions on the left lower limb associated 2 melanomas.	Segmental (Blaschko's lines broad bands)	No
	49	F	No	3	#1-3: SSM.	Yes	Multiple lentigines on the left arm associated with 3 melanomas.	Segmental (Blaschko's lines broad bands)	No
Synchronous melanomas arising within nevus spilus^13^	83	M	No	4	#1: SSM, BD: 2.51 mm. #2: SSM, BD: 1.18 mm. #3: MIS.	Unspecified	Light tan patch with speckled lentiginous pigmentation on the external aspect of his right arm and forearm associated 4 melanomas.	Segmental (Blaschko's lines broad bands)	Yes
Melanoma arising in segmental nevus spilus^6^	42	M	N/A	1	MM, BD: 0.6 mm.	Yes	Multiple pigmented lesions with underlying tan patch on his abdomen, left side and lower back associated 1 melanoma.	Segmental (Blaschko's lines broad bands)	No

*BD*, Breslow depth; *F*, Female; *FHM*, Family history of melanoma; *M*, male; *MIS*, melanoma in situ; *MM*, malignant melanoma; *SSM*, superficial spreading melanoma.

Given the diversity of commonly observed cutaneous melanocytic proliferations and neoplasms and the underlying genetic changes, variable clinical presentations of CMMo are not surprising. Mosaicism can arise due to de novo post-zygotic mutation in 1 allele of a specific gene during cell migration and organogenesis. This process leads to an altered gene and the emergence of both affected and normal cell populations. Alternatively, when mosaicism arises in individuals with an autosomal dominant disease, a subsequent post-zygotic mutation may deactivate the previously normal allele, triggering the clinical presentation of pathology in a mosaic pattern.^2^ Although mosaicism associated with malignant tumor development often arises from a second hit in the previously unaffected allele of the responsible gene, it is possible that crucial genetic changes in melanoma may require mutations of pathways not involved in establishing the mosaic variant observed in nevi. Thus, mutations in some CMMo may not increase melanoma risk or only minimally enhance melanoma formation, while in other cases, the CMMo may show a much higher risk for melanoma. Additionally, CMMo may arise later in life only after mutations accumulate in the mosaic areas or due to delayed activation and migration of dormant melanocytes influenced by unknown migration factors.^5^

The increased melanoma risk in these CMMo highlights the potential role of somatic mutations in enhancing malignant tumor development and suggests that genetic and epigenetic studies of CMMo may provide an unparalleled insight into melanomagenesis. This helps clinicians focus surveillance on high-risk mosaic areas and educate patients on targeted skin self-examinations. Further research to clarify the role of the somatic mutations driving malignancy in CMMo patients is needed. Genetic research is crucial as it can shed light on the mechanisms behind melanoma development and enhance our comprehension of melanoma pathogenesis overall. By improving recognition of CMMo-associated melanoma as a distinct entity, future research and clinical strategies can be better tailored to detect and manage these high-risk patients.

## Conclusions

Recognition of mosaic patterns highlights the heightened melanoma risk in affected regions and supports a lower threshold for biopsy when evaluating even subtly irregular lesions. Complementary patient education is essential and may improve adherence to self-skin checks focused on mosaic regions. Additionally, genetic studies on CMMo will help elucidate the underlying mechanisms driving melanocytic nevus formation and oncogenic senescence and may uncover novel aspects of melanomagenesis. Prior case reports have employed varied terminology, hindering further research on its role in melanoma pathogenesis. By increasing awareness and unifying the variety of clinical manifestations of this phenomenon, we strive to identify more patients with CMMo-associated melanomas, igniting interest in advancing this research further.

## Conflicts of interest

None disclosed.
